# Emerging Super-specialty of Neurology: Intraoperative Neurophysiological Monitoring (IONM) and Experience in Various Neurosurgeries at a Tertiary Care Hospital in Doha, Qatar

**DOI:** 10.7759/cureus.20432

**Published:** 2021-12-15

**Authors:** Liaquat Ali, Faisal R Jahangiri, Arshad Ali, Sirajeddin Belkhair, Osama Elalamy, Gholam Adeli, Mohammad Alghazow, Rakesh Krishnan, Fazal Karim, Ambreen Iqrar, Ali Raza

**Affiliations:** 1 Department of Neurology, Neuroscience Institute, Hamad General Hospital, Doha, QAT; 2 School of Behavioral and Brain Sciences, The University of Texas at Dallas, Richardson, USA; 3 Intraoperative Neuromonitoring Program, Labouré College of Healthcare, Milton, USA; 4 Neurophysiology, Axis Neuromonitoring, Richardson, USA; 5 Neurophysiology, Global Innervation, Dallas, USA; 6 Department of Neurosurgery, Neuroscience Institute, Hamad General Hospital, Doha, QAT; 7 Department of Clinical Academic Sciences, College of Medicine, Qatar University, Doha, QAT; 8 Department of Neurological Sciences, Weill Cornell Medicine, Doha, QAT; 9 Department of Neurology, Aga Khan Health Service, Pakistan, Karachi, PAK

**Keywords:** orthopedic, neurosurgery, multimodality ionm, somatosensory evoked potentials (ssep), transcranial electrical motor evoked potential (tcemep), electromyography, emg, ionm, neuromonitoring, neurophysiology

## Abstract

Introduction

Intraoperative neurophysiological monitoring (IONM) helps in better patient outcomes by minimizing risks related to the functional status of the nervous system during surgical procedures. An IONM alert to the surgical team during the surgery can help them identify the cause and take immediate corrective action. IONM confers possible benefits, including improved surgical morbidity and mortality, better patient care, minimal neurological deficits, reduced hospital stay, medical costs, and litigation risk. In addition, a highly skilled IONM team will make a better patient outcome.

Methods

We retrospectively reviewed 62 consecutive patients who underwent intracranial and spinal neurosurgical procedures. Multimodality IONM was utilized, including somatosensory evoked potentials, transcranial electrical motor evoked potential, spontaneous and triggered electromyography, electroencephalography, electrocorticography, cortical sensory mapping, and direct electrical cortical stimulation. Of a total of 62 patients, two patients revealed neurotonic EMG discharges during IONM, and most patients woke up without any new neurological deficit.

Results

Sixty-two patients were included, ranging from age 5 to 77 years (mean 43.5 years), with 54.8% men and 45.2% female. Multimodality IONM was used in all patients. Two EMG alerts were recorded during IONM, during a brain tumor resection, and right acetabular hip surgery with postoperative right foot drop.

Conclusion

Multimodality IONM is the gold standard of care for any surgical services and is used as real-time monitoring of functional integrity of neural structures at risk. If utilized by trained and expert teams, numerous surgeries may benefit from multimodality intraoperative neurophysiologic monitoring.

## Introduction

Today, intraoperative neurophysiological monitoring (IONM) has become the gold standard of care in most hospitals that provide neurological, orthopedic, vascular, and cardiothoracic surgical services. The use of IONM can decrease the risk of paralysis and other complications during critical procedures [1-3]. In addition, IONM allows surgeons to know the neurologic status of a patient throughout the surgical procedure.

IONM consists of a diversity of neurophysiological tests known as modalities. For example, somatosensory evoked potentials (SSEP), transcranial electrical motor evoked potential (TCeMEP), spontaneous and triggered electromyography (sEMG/tEMG), nerve action potential (NAP), train of four (TOF), brainstem auditory evoked potentials (BAEP), visual evoked potentials (VEP), electroencephalography (EEG), electrocorticography (ECoG), cortical sensory and motor mapping. During surgeries, IONM helps in evaluating the functional integrity of the peripheral and central nervous systems in real-time. It alerts the surgeon to possible neurologic injury and prompts corrective measures to prevent potential permanent disability, thus improving surgical outcomes [4]. During neurosurgery procedures, these modalities help to monitor the functional integrity of specific neural structures like nerves, spinal cord, and brain parts.

IONM aims to lower the risk of operative and postoperative neurological deficits during high-risk neuro, orthopedic, otolaryngology (ENT), and vascular surgeries, as well as interventional procedures. IONM can minimize impending damage to vital nervous system structures (brain, brainstem, spinal cord, nerves) and alert the surgeon to this potential damage to take an intraoperative corrective action. IONM confers possible benefits, including improved surgical morbidity and mortality, better patient care, minimal neurological deficits, reduced hospital stay, medical costs, and litigation risk. In addition, a highly skilled IONM team will make a better patient outcome [5]. The presence of a certified technologist (Certification in Intraoperative Neurophysiological Monitoring-CNIM) [6], board-certified neurophysiologist (DABNM) [7], or neurologist (ABCN-IONM) [8-9] on-site or remotely will have a better outcome than non-certified teams. IONM is the standard procedure for any neurosurgical intervention in the world. We recently started IONM locally and reviewed our local experience and how to improve the quality of IONM and reduce the risk of insult of neuronal structures of the brain and spinal cord.

## Materials and methods

The study’s objective was to determine the frequency of neurological injury or insult during various neurosurgical procedures under IONM [10]. This is an observational cross-sectional retrospective study of chart/data review of 62 patients operated between January 01, 2017 and March 01, 2020, at Hamad General Hospital Doha, Qatar. All patients with clinical neurologic deficits documented preoperatively, postoperatively, and radiologically (MRI/CT head and spine) diagnosed with brain and spine lesions were enrolled. Patient charts were reviewed by an experienced surgical intraoperative neurophysiologist and neurologist. Primarily, total intravenous anesthesia (TIVA) was used in all procedures. During IONM, multiple monitoring modalities were utilized, such as somatosensory evoked potentials (SSEP), motor evoked potentials (MEP), brainstem auditory evoked potentials (BAEP), electromyography (free-running and triggered EMG), nerve action potential (NAP), electroencephalography (EEG), electrocorticography (ECoG), sensory mapping, and direct electrical cortical stimulation (DECS). In addition, all patients who underwent IONM for various neurosurgical spine and brain intervention procedures were included. The data recorded on a structured data sheet had demographic, clinical diagnosis, radiologic findings, name of surgical intervention, IONM alert to the surgeon, changes in signals, anesthesia, and postoperative discharge follow-up.

Descriptive statistics were used to summarize and determine the sample characteristics and distribution of various considered parameters related to demographic, diagnostic, clinical features and related outcome measures, and other related features of the patients. The data and results were reported with mean and standard deviation (SD) with corresponding 95% CI; the remaining results were reported with median and interquartile range (IQR). Frequencies and percentages were used for summarizing the categorical data. As appropriate, associations between two or more qualitative variables were examined and assessed using Pearson’s chi-square and Fisher’s exact tests. Unpaired t-test and ANOVA were used to compare the mean values of different quantitative parameters between two or more groups. Pearson or Spearman rank-order correlation was used to quantitatively evaluate the correlation between various outcomes. A two-sided P value <0.05 was statistically significant. All statistical analyses were done using statistical packages SPSS 24.0 (SPSS Inc., Chicago, IL) and Epi Info 2000 (Centers for Disease Control and Prevention, Atlanta, GA). This study was approved by Medical Research Center Hamad Medical Corporation, Doha, Qatar (MRC-01-20-137) on August 26, 2020.

Intraoperative neurophysiological monitoring (IONM) modalities

Somatosensory Evoked Potentials (SSEP)

SSEP assess the integrity of large nerve fibers and spinal dorsal column-medial lemniscus sensory system. SSEP may be attained by direct electrical stimulation of peripheral nerves (e.g., median, ulnar, tibial, peroneal, and saphenous nerves, etc.) and recorded at various levels within the neuraxis such as peripheral nerves, spinal cord, and brainstem somatosensory cortex. The responses are compared with normal laboratory values and the patient’s baseline recordings. It is essential to obtain established reproducible baseline recordings before any change in the position of patients or surgical interventions. The most critical indicator of neurological injury or dysfunction is any changes from baseline recorded responses. Anesthetic inhalational and intravenous agents, mean arterial pressure (MAP), temperature, etc., may affect the intraoperative neurophysiological data, and it is vital to monitor them [11]. An increase in latency of more than 10% and/or a decrease in more than 50% amplitude is considered an alert for SSEP [3]. SSEP may be utilized in a variety of neurosurgical, vascular, and orthopedic interventions.

Motor Evoked Potentials (MEPs)

MEPs help in protecting the corticospinal tracts during brain, brainstem, and spine surgeries. MEPs are sensitive to most anesthetics agents (inhalational and intravenous) and neuromuscular blockers. Intraoperative MEP can be elicited by transcortical (TCeMEP) or direct cortical electrical stimulation (DECS) of the brain. The responses are recorded over the spinal cord (as Direct D wave and indirect I wave) and from muscles as compound muscles action potentials (CMAP) [12]. An alert for MEP is either complete loss or abrupt, a significant decrease in amplitude of 70-80% without an explanation. Systemic factors or an anesthetic fade phenomenon have a more gradual effect on MEP signals [13]. A change in waveform morphology or increase of 100 volts or more stimulation threshold may be considered an alert. Any surgical risk to the corticospinal (motor) tract is an indication for MEP monitoring. The MEPs can be utilized in a wide variety of surgical procedures such as spinal cord surgeries, scoliosis, tethered cord release, cauda equina surgeries, brain, brainstem surgeries, and vascular surgeries such as descending aortic and spinal arteriovenous malformation (AVM), carotid endarterectomy, and hip surgeries, etc. Safety concerns reported during MEP monitoring may include bite injuries, thermal injury of the brain or scalp, seizures, arrhythmias, and movement-induced injuries. Specific precautions must be considered in patients with cochlear implants, deep brain stimulation (DBS), pacemaker and epilepsy, etc. [14]. A multimodality approach with MEP, SSEP, and EMG gives better protection than single modality monitoring.

Spontaneous and Triggered Electromyography (EMG)

Spontaneous EMG (sEMG) allows real-time assessment by recording spontaneous muscle activity. Free-running sEMG detects any surgical, mechanical irritation to peripheral or cranial nerves before irreversible damage occurs. Triggered electromyography (tEMG) is performed by applying electrical stimulation directly to the brain, brainstem, spinal cord, or nerve and recording a CMAP response. Thus, this may be used as a mapping tool to detect the location of peripheral or cranial nerves that may be difficult to distinguish from the tumor, filum, and fatty tissues during surgical resections. Triggered EMG may also be used to confirm the functional integrity of the nervous tissue. Thus, EMG is useful during various neurosurgical, orthopedic, and ENT procedures [15-16].

Brainstem Auditory Evoked Potentials (BAEPs)

The auditory pathways can be monitored intraoperatively by short-latency BAEPs. To record BAEPs responses, auditory clicks stimulation in the ear are used, and these clicks are broadband sound range (500 to 4000 Hz) to deliver at various audio frequencies [17-19]. The most common causes of surgical injuries to the auditory system are surgical compression, traction, thermal and ischemic injuries. Sudden loss of all BAEPs waves may be due to ischemia of the cochlea from trauma to the internal auditory artery. BAEP is resistant to anesthetics, including volatile agents, but hypothermia has a significant effect with delay in latencies [4]. BAEP may dramatically change in neonates and infants before the age of two years. The alert criteria for BAEP is either decrease in wave V amplitude or an increase in wave I-III, III-V, or I-V interpeak latencies (IPL). An increase in 0.5 ms wave I-V IPL is an alert, and intervention must be done with more than a 1.0 ms increase in IPL to avoid postoperative hearing loss. A persistent decrease in the wave V amplitude or wave I-V IPL is predictive of postoperative hearing loss.

## Results

Sixty-two patients were admitted for various neurosurgical or orthopedic interventions and for IONM between January 01, 2017 and March 01, 2020, in Hamad General Hospital Doha, Qatar. The ages ranged from 5 to 77 years (mean 43.5 years), 54.8% were men (34/62), and 45.2% were female (28/62) (Table 1).

**Table 1:** Demographic distributions of the patients

Age (years)	Gender	Frequency	Percentage (%)
Mean=43.5	Male	34	54.8%
	Female	28	45.2%

Of all these patients, the underlying diagnosis was brain tumors 48.4% (including meningiomas, astrocytoma, glioblastoma multiforme, oligodendroglioma, medulloblastoma, pontine ganglioglioma, metastasis, epidermoid cysts), brain cavernoma/AVM 16.4%, lumbar spinal stenosis 11.3%, nerve sheath tumors/schwannoma 9.7%, spinal cord tumors 8.1%, spinal vertebral fracture 8.1%, and spinal scoliosis 1.6% (Table 2).

**Table 2:** Preoperative diagnosis AVM: arteriovenous malformation

Diagnosis				Frequency	Percentage
Intracranial brain tumors+metastasis+cysts				30	48.4%
Brain cavernoma/AVM				10	16.4%
Lumbar spinal stenosis				7	11.3%
Nerve sheath tumor				6	9.7%
Spinal vertebral fracture				5	8.1%
Spinal cord tumor				5	8.1%
Spinal scoliosis				1	1.6%

During IONM, different modalities were utilized (Table 3). The multimodality approach, including SSEP+MEP+EMG, was 29%, SSEP+EMG was 17.7%, SSEP+MEP was 16.1%, SSEP+MEP+EMG+EEG was 11.3%, SSEP+MEP+EMG+BAEP was 6.4%, and electrocorticography (ECoG) and direct cortical stimulation were 3.2%. In addition, there were two alerts to the surgeon during IONM as shown in Table 4, including EMG neurotonic discharges in glossopharyngeal muscles in tentorial meningioma surgery and in tibialis anterior and extensor hallucis longus muscles during right acetabular hip fracture of open reduction and internal fixation (ORIF) surgery with postoperative right foot drop. Primarily TIVA with no muscle relaxation after intubation was used in IONM.

**Table 3:** Modalities Intraoperative neurophysiology monitoring (IONM) modalities used. SSEP: somatosensory evoked potentials; MEP: motor evoked potentials; EMG: electromyography; EEG: electroencephalography; BAEP: brainstem auditory evoked potentials; DECS: direct electrical cortical stimulation

IONM Modalities		Frequency	Percentage (%)
SSEP+MEP+EMG		18	29%
SSEP+EMG		11	17.7%
SSEP+MEP		10	16.1%
SSEP+MEP+EMG+EEG		7	11.3%
SSEP+MEP+EMG+BAEP		4	6.4%
DECS		2	3.2%

**Table 4:** Intraoperative alerts recorded EMG: electromyography

Alerts			Frequency	Percentage
	Neurotonic EMG discharges glossopharyngeal muscles		1	1.6%
	Neurotonic EMG discharges right sciatic innervated muscles- post-op foot drop.		1	1.6%

## Discussion

IONM performed in real-time is a gold standard of care that provides protection to the central and peripheral nervous system during neurosurgical, orthopedic, vascular, ENT, interventional, and cardiothoracic procedures [2, 20]. This alerts the surgeons to potential neurologic injury and urgent corrective measures to prevent permanent neuronal tissues injury, thus improving surgical outcomes. In this study, the most common underlying diagnoses were brain tumors (48.4%) and brain cavernoma/AVM (16.4%). The most common IONM modalities used were SSEP, MEP, and EMG (29%). One patient showed abnormal neurotonic EMG discharges in the cranial nerve during neuromonitoring, and the other showed right sciatic nerve with postoperative foot drop. Identification of abnormal EMG responses and immediately reporting to the surgeon helped in taking the corrective action. Our study showed that a multimodality IONM during different surgical procedures could prevent devasting neurologic insults of neural structures at risk.

Bhagat et al.’s (2015) retrospective review of 354 consecutive patients who underwent spinal deformity surgery demonstrated the superiority of combined multimodality IONM over either single modality in early detection of impending neurological injuries. In addition, the overall sensitivity and specificity of combined SSEPs and MEPs were found to be 100% and 99.3%, respectively, strongly supporting its use [21]. Thirumala et al. (2016) reported in a meta-analysis with seven studies including 2052 patients with idiopathic scoliosis (IS). The incidence of neurological deficit was 0.93%. The pooled sensitivity, specificity, and diagnostic odds ratio were 82.6% (95% CI: 56.7%-94.5%), 94.4% (95% CI: 85.1%-98.0%), and 106.16 (95% CI: 24.952-451.667), respectively [22]. In another retrospective study including 296 patients by Neira et al. (2016), 51 (17%) patients had IONM alerts, 41 were only TCeMEP, five were only SSEP, and five were in both modalities. The sensitivity was estimated to be 93.5%, 92.2%, and 46.7% for TCeMEPs, combination (either TCeMEPs or SSEPs), and SSEPs, respectively. TCeMEPs are more sensitive than SSEP at detecting impending new neurological deficits [23].

Ishida et al. (2019) reported diagnostic and therapeutic values of IONM during resection of intradural extramedullary spinal tumors. In predicting neurological deficits at the 6-month follow-up, IONM yielded a sensitivity of 82.4%, specificity of 90.7%, positive predictive value (PPV) of 63.6%, and negative predictive value (NPV) of 96.3% [24]. Pérez-Sanpablo et al. (2017) evaluated the monitoring rate, sensitivity, and specificity of IONM during removal of intradural extramedullary (IDEM) or epidural metastatic spinal tumors. The sensitivity, specificity, and predictability of TCeMEP for motor changes were 93%, 90%, and 91%, respectively. Conversely, the sensitivity, specificity, and predictability of SSEP were 62%, 97%, and 89%, respectively. Thus, MEP shows higher sensitivity than SSEP does [25].

There were no false-negative or false-positive data in this study. There was one true-positive case where the patient woke up with a foot-drop. The limitation of this study was a small number of surgical procedures as various surgical departments are beginning to utilize the IONM in our hospital.

## Conclusions

This single-center review during a wide variety of surgeries shows the importance of multimodality IONM. The incidence of alerts was low, but the total number of surgical procedures were also small. Each modality has its benefit and limitations but, when used in combination, gives better protection to the patient. Numerous types of surgeries may utilize and benefit from multimodality intraoperative neurophysiologic monitoring. In addition, better patient outcomes are dependent on the experience of surgical and neurophysiological monitoring teams. Multimodality intraoperative neurophysiologic monitoring is the gold standard of care for many surgical services and should be used for real-time monitoring of functional integrity of neural structures at risk.
